# Effects of ginkgo leaf extract on growth performance, nutrient utilization, blood biochemical parameters, meat quality, fatty acid profiles, and gene expression in growing rabbits

**DOI:** 10.1093/jas/skaf395

**Published:** 2025-11-06

**Authors:** Qi Lu, Rui Chen, Shuanglong Xie, Xu Wang, Jixiao Qin, Yiqing Xu, Yiming Ban, Chengcheng Gao, Peiyao Li, Xingzhou Tian

**Affiliations:** Key Laboratory of Animal Genetics, Breeding and Reproduction in the Plateau Mountainous Region, Ministry of Education, College of Animal Science, Guizhou University, Guiyang, 550025, China; Key Laboratory of Animal Genetics, Breeding and Reproduction in the Plateau Mountainous Region, Ministry of Education, College of Animal Science, Guizhou University, Guiyang, 550025, China; Key Laboratory of Animal Genetics, Breeding and Reproduction in the Plateau Mountainous Region, Ministry of Education, College of Animal Science, Guizhou University, Guiyang, 550025, China; Key Laboratory of Animal Genetics, Breeding and Reproduction in the Plateau Mountainous Region, Ministry of Education, College of Animal Science, Guizhou University, Guiyang, 550025, China; Key Laboratory of Animal Genetics, Breeding and Reproduction in the Plateau Mountainous Region, Ministry of Education, College of Animal Science, Guizhou University, Guiyang, 550025, China; Key Laboratory of Animal Genetics, Breeding and Reproduction in the Plateau Mountainous Region, Ministry of Education, College of Animal Science, Guizhou University, Guiyang, 550025, China; Key Laboratory of Animal Genetics, Breeding and Reproduction in the Plateau Mountainous Region, Ministry of Education, College of Animal Science, Guizhou University, Guiyang, 550025, China; Key Laboratory of Animal Genetics, Breeding and Reproduction in the Plateau Mountainous Region, Ministry of Education, College of Animal Science, Guizhou University, Guiyang, 550025, China; Key Laboratory of Animal Genetics, Breeding and Reproduction in the Plateau Mountainous Region, Ministry of Education, College of Animal Science, Guizhou University, Guiyang, 550025, China; Key Laboratory of Animal Genetics, Breeding and Reproduction in the Plateau Mountainous Region, Ministry of Education, College of Animal Science, Guizhou University, Guiyang, 550025, China

**Keywords:** ginkgo leaf extract, growth performance, antioxidant activity, meat quality, rabbit

## Abstract

The objective of this study was to observe the effects of ginkgo leaf extract (GLE) on growth performance, nutrient utilization, antioxidant activity, carcass yield, meat quality, fatty acid profiles, and gene expression in rabbits. A total of 80 weaned male rabbits were randomly divided into 4 equal treatment groups according to a completely randomized design. The control (CON) group was fed a basal diet, whereas treatments 1 (LG), 2 (MG), and 3 (HG) were fed a basal diet supplemented with 2, 4, and 6 g/kg GLE, respectively. The results indicated that compared with the CON group, the MG treatment group presented significantly (*P *< 0.05) greater body weight change and average daily gain from 0–42 d. The mortality rate was significantly lower (*P *< 0.05) in rabbits fed GLE than in those of the CON group. Additionally, the apparent digestibility of acid detergent fibre, hemicellulose, and ash in the rabbits in the MG group was greater (*P *< 0.05) than that in the CON group. Compared with the CON group, dietary supplementation with GLE significantly increased nitrogen retention. Compared with those in the other three treatment groups, the gross energy (GE) absorption and GE retention in the MG treatment group significantly increased (*P *< 0.05). Compared with the CON group, the GLE-supplemented groups presented significantly (*P *< 0.05) increased plasma catalase levels and significantly (*P *< 0.05) decreased malondialdehyde and total cholesterol contents. Compared with those fed the CON diet, rabbits fed the LG and MG diets presented decreased (*P *< 0.05) levels of yellowness, drip loss, and shear force in the muscle. Compared with the CON diet, the addition of GLE significantly (*P *< 0.05) decreased C16:0 and C20:4n6 levels, whereas it significantly (*P *< 0.05) increased C18:3n3 and n-3 polyunsaturated fatty acid (PUFA) levels. Compared with the CON diet, GLE supplementation significantly increased (*P *< 0.05) nuclear factor (erythroid-derived 2)-like 2, haem oxygenase 1, superoxide dismutase 1, and glutathione peroxidase 1 levels, whereas it significantly decreased (*P *< 0.05) kelch-like ECH-associated protein 1 levels. Taken together, these findings suggest that dietary supplementation with GLE could improve growth performance, nutrient utilization, meat quality, n-3 PUFA concentrations, and antioxidant activity in growing rabbits. The optimum level of GLE supplementation in the diet for growing rabbits was 4 g/kg under the conditions of this experiment.

## Teaser

Feeding a flavonoid-rich ginkgo leaf extract improves antioxidant activity and meat quality in rabbits.

## Introduction

Oxidative stress (OS) is the most important cause of cell damage, leading to the occurrence and development of many diseases in rabbits and consequently impairing physiological functions (Mutwedu et al., 2021). Saghir et al. (2023) reported that OS can reduce the feed conversion rate, growth performance, and antioxidant activity of meat rabbits. Notably, many other studies have reported that natural antioxidants (i.e., flavonoids) can increase blood total antioxidant capacity (TAC) and glutathione (GSH) concentrations and decrease malondialdehyde (MDA) contents, thereby increasing antioxidant potential and alleviating OS in rabbits (El-Sheakh et al., 2015; El-Ratel et al., 2025). For example, Liu et al. (2011) reported that dietary supplementation with 10 g/kg natural extract of chestnut wood had a positive effect on the production performance and antioxidant potential of male New Zealand rabbits. Thus, antioxidants can effectively prevent and eliminate the imbalance in redox balance caused by OS and improve growth performance, carcass quality, and antioxidant activity in rabbits (Abd et al., 2024).

Flavonoid compounds are natural antioxidants that widely exist in nature; their antioxidant activity is related to their ability to modulate oxidation pathways, eliminate free radicals, and form metal-ion chelates (Kamada et al., 2005; Zhan et al., 2017). Sheweita et al. (2025) reported that dietary supplementation with 20 mg/kg gallic acid could enhance antioxidant function in male rabbits by increasing the levels of superoxide dismutase (SOD), glutathione s-transferase, glutathione reductase, GSH, and glutathione peroxidase (GPX) in testis tissues. In addition, a previous study revealed that the activation of the nuclear factor (erythroid-derived 2)-like 2 (Nrf2) signalling pathway could induce endogenous increases the antioxidant enzymes, such as SOD, GPX, and catalase (CAT), and thus enhance antioxidant functions in animals (Levonen et al., 2007). Notably, flavonoid compounds, such as gallic acid, might disturb protein-protein interactions between kelch-like ECH-associated protein 1 (Keap1) and Nrf2 and subsequently increase the expression of Nrf2 and its various downstream antioxidant genes (Feng et al., 2018). Wang et al. (2025) suggested that supplementation with flavonoid-rich plant extract could increase the abundance of Nrf2 and its downstream antioxidant genes SOD, GPX, and CAT in muscle and subsequently increase the antioxidant status of rabbits.

In addition, various studies have indicated that the administration of feed supplemented with flavonoid plant extracts to animals improved growth performance and meat quality (Ouyang et al., 2016; Selim et al., 2021). For example, North (2019) reported that dietary supplementation with 2 g/kg quercetin could improve the nutritional quality of meat by increasing the polyunsaturated fatty acid (PUFA) content in the *longissimus thoracis et lumborum* (LTL) muscle in growing rabbits. Similarly, Wang et al. (2025) reported that 1 g/kg *Houttuynia cordata* Thunb. extract improved meat quality and upregulated LTL muscle antioxidant gene expression in weaned rabbits.

Gingko leaves are rich in naturally occurring bioactive compounds, such as flavonoid compounds, that may improve the health of the body in rabbits (Khaleel and Ali, 2018). Interestingly, gingko leaf extract (GLE), a substance with different biological functions that can inhibit tumour necrosis factor-alpha-induced reactive oxygen species generation, increase the level of GSH, decrease the content of MDA, and enhance the antioxidant functions, has drawn widespread attention given its application in animal husbandry (Hussein et al., 2017). For instance, Mustafa and Hussein (2023) reported that GLE contains an abundance of flavonoid compounds and that GLE could increase the concentrations of serum immunoglobulin A, immunoglobulin G and immunoglobulin M, and the antioxidant enzymes TAC, SOD, GPX, and CAT in New Zealand White rabbits. However, research on growth performance and meat quality when GLE is used as a rabbit dietary supplement is limited. We hypothesized that dietary supplementation with GLE could improve the growth performance, meat quality, and antioxidant function of the LTL muscle in rabbits. Therefore, the objective of the present study to investigate the effects of dietary supplementation with GLE on the growth performance, nutrient utilization, blood biochemical parameters, carcass yield, relative organ weight, meat quality, fatty acid profiles, and gene expression of weaned rabbits.

## Materials and Methods

### Animals and experimental design

This study was conducted in accordance with and was reviewed by the Subcommittee of Experimental Animal Ethics of Guizhou University (No. EAE-GZU-2024-E025, Guiyang, China). A total of 80 weaned (35 d) New Zealand White male rabbits with similar body weights (BWs, mean ± standard deviation, 1071.06 ± 83.58 g) were randomly divided into 4 equal treatment groups according to a completely randomized design. The control (CON) group was fed a basal diet, and treatments 1 (LG), 2 (MG), and 3 (HG) were fed a basal diet supplemented with 2, 4, and 6 g/kg GLE, respectively. Each treatment included 20 rabbits, which had 10 replications (*n *= 10), with 2 rabbits per replicate (2 rabbits per cage). The basal diet contained 235 g/kg corn, 65 g/kg soybean meal, 30 g/kg rapeseed meal, 32 g/kg cottonseed meal, 166 g/kg wheat bran, 422 g/kg alfalfa, 40 g/kg compound-premix, 5 g/kg calcium hydrogen phosphate, 3 g/kg salt, and 2 g/kg stone powder. The four groups of diets had the same basal diet, but they had different GLE levels. The GLE was purchased from Guosheng Biotechnology Co., Ltd (Shaanxi, China), and the flavonoid compounds of GLE are shown in Table 1. All rabbits were individually housed in polyvalent wire-mesh cages (160 × 70 × 195 cm) during the experimental feed period. The rabbits were fed a pelleted diet generated using a 4 mm mill in a granule presser (Jixiang Animal Husbandry Machinery Co., Ltd, Henan, China), and feed was offered twice daily at 08:00 and 17:00. Feed and water were provided *ad libitum*. The feeding trial period lasted 49 d, which included a 7-day adaptation period and a 42-day formal experimental period. Moreover, a two-phase formal experimental period was used: 0–21 d and 21–42 d. The nutrient requirements met the Chinese standard NY/T 4049–2021, and the ingredients and chemical composition of the basal diet are shown in Table 2.

**Table 1.** Flavonoid compounds of ginkgo leaf extract

Item^1^	Flavonoid compounds, μg/kg of DM
**Gallic acid**	157.60 ± 12.71
**Protocatechuic acid**	910.59 ± 96.93
**Protocatechualdehyde**	nd^2^
**Chlorogenic acid**	nd
**Catechin**	27.08 ± 10.16
**Caffeic acid**	41.69 ± 4.22
**Epicatechin**	nd
**Homoorientin**	13.49 ± 1.16
**Vitexin-2-o-rhamnosid**	nd
**Rutin**	nd
**Neohesperidin**	19.48 ± 6.57
**Isorhamnetin-3-o-neohespeidoside**	37.66 ± 19.17
**Isovitexin**	nd
**Vitexin**	nd
**Hyperoside**	nd
**Kaempferol 3-rutinoside**	131.14 ± 29.43
**Rhoifolin**	267.22 ± 18.75
**Naringin**	46.29 ± 1.76
**Hesperidin**	nd
**Genistin**	151.83 ± 19.59
**Umbelliferone**	nd
**Tiliroside**	23.99 ± 2.14
**Resveratrol**	nd
**Quercetin**	47.94 ± 0.88
**Apigenin**	53.48 ± 22.68
**Naringenin**	4.26 ± 0.54
**Genistein**	nd
**Kaempferol**	71.27 ± 20.99
**Psoralen**	nd
**Aurantio-obtusin**	nd
**Bergapten**	nd
**Asiatic acid**	nd
**Rhein**	nd
**Aloe-emodin**	nd
**Nobiletin**	5.57 ± 0.20
**Galangin**	nd
**Emodin**	14.07 ± 0.12
**Chrysophanol**	nd
**Physcion**	nd
**Ursolic acid**	4.95 ± 2.71

1 Values represent the means of three replicates (*n *= 3). mean ± standard deviation.

2 nd, not detected.

**Table 2.** The ingredients and chemical composition of basal diet in this study

Ingredient	Content, g/kg	Chemical composition^2^	Content, g/kg of DM^1^
**Corn**	235	DM, g/kg of the as-fed diet	891
**Soybean meal**	65	OM	901
**Rapeseed meal**	30	GE, KJ/g	154
**Cottonseed meal**	32	CP	162
**Wheat bran**	166	NDF	346
**Alfalfa**	422	ADF	212
**Compound-premix^3^**	40	Hemicellulose	134
**Calcium hydrogen phosphate**	5	Ash	99
**Salt**	3		
**Stone powder**	2		

1 Values represent the means of three replicates (*n *= 3).

2 DM, dry matter; OM, organic matter; GE, gross energy; CP, crude protein; NDF, neutral detergent fibre; ADF, acid detergent fibre.

3 Compound-premix was contained of kg: 150 KIU of V_A_, 10 KIU of VD_3_, 300 IU of VE, 20 mg of VK, 300 mg of VB_3_, 200 mg of calcium pantothenate, 4 mg of folic acid, 1 mg of biotin, 300 mg of Cu, 1500 mg of Fe, 1500 mg of Zn, 500 mg of Mn, and 3.5 mg of I.

### Chemical composition and flavonoid compounds

At the end of the experiment, the feed and faeces were dried in an air-circulating oven (WGL-125D; Test Instrument Co., Ltd, Tianjin, China) at 105 °C for 48 h to determine the dry matter (DM) content according to method 934.01 of the Association of Official Analytical Chemists (AOAC; 2005). The crude protein (CP)/nitrogen (N) ratios of the feed, faeces, and urine were analysed using the Kjeldahl method (CP = factor of 6.25 × N; 988.05). The feed and faeces were dried at 550 °C in a muffle furnace (TNX140030; Shanghai Shinbae Industrial Co., Ltd, Shanghai, China) to determine the ash content (942.05). The neutral detergent fibre (NDF) and acid detergent fibre (ADF) contents of the feed and faeces were determined using a Fibertherm machine (Gerhardt, Bonn, Germany) according to the methods of AOAC 2002.04 and AOAC 973.18, respectively. The organic matter (OM) content was calculated as 100-ash, and the hemicellulose content was calculated as NDF-ADF. The gross energy (GE) of the feed, faeces, and urine was determined using an adiabatic oxygen bomb calorimeter.

Moreover, the flavonoid compounds of GLE were identified using high-performance liquid chromatography and tandem mass spectrometry (HPCL/MS) according to the methods of Wang et al. (2024). The HPLC conditions were as follows: mobile phase A was acetonitrile; mobile phase B was 0.1% formic acid; a diode array detector was used; the column temperature was 35 °C; the injection temperature was 4 °C; the injection volume was 2 μL; and the flow rate was 0.3 mL/min. The MS conditions were as follows: the aux gas pressure was 70 psi, the nebulization temperature was 350 °C, and the ion spray voltage was 4500 V.

### Growth performance and mortality rate

The amount of feed supplied to the rabbits and leftovers was used to calculate the amount consumed, and the average daily feed intake (ADFI) was calculated at the end of the experiment. The BW of each animal was recorded at 0 d, 21 d, and 42 d during the formal experimental period to calculate the BW change (BWC), ADG, and FCR according to Li et al. (2023) and Wang et al. (2024). ADFI (g/d) = (feed quantity-surplus)/d; BWC (g) = final weight-initial weight; ADG (g/d) = BWC/d; FCR = ADFI/ADG. In addition, the mortality rate of the rabbits, including the number of dead rabbits of each experimental type, was observed and recorded every day. The mortality rate was calculated according to the following equation (Wang et al., 2018): mortality rate (%) = (number of dead rabbits/number of rabbits) ×100%.

### Nutrient utilization

A digestion and metabolism experiment was carried out at the end of 7 d, which consisted of a 2-d adaptation period and a 5-d sample collection period. The total faeces and urine in each cage were collected daily. The faecal and urine samples were stored at −20°C until the end of the experiment. The faecal samples were divided into two parts. One part was acidified with 10% sulfuric acid (H_2_SO_4_; 100 g was added to 10 mL) to determine N, and the second part consisted of fresh faeces for determining the composition of other chemicals. The faecal samples were oven-dried at 65 °C to determine the moisture content, and representative samples of dried faeces were taken for proximate analysis. The urine sample was also divided into two parts. One part consisted of fresh urine to detect GE, and the second part was acidified with 10% H_2_SO_4_ to maintain a pH < 3 and kept in a container to determine N.

### Plasma lipid metabolism and antioxidant activity parameters

The rabbits were raised to 84 days of age, at which time one rabbit was randomly selected from each cage. A total of 40 rabbits were fasted for 12 h. All rabbits were anaesthetized by intramuscular injection of ketamine (1.5 mg/kg of BW) and xylazine (10 mg/kg of BW). Next, a 5 mL volume of autologous blood was obtained from each rabbit via cardiac puncture using a sodium heparin vacuum tube. During collection, the blood collection needle was fixed, and the vacuum tube was rotated intermittently to prevent clotting. The blood sample was centrifuged at 4,000 ×*g* for 10 min at 4 °C, after which the supernatant was collected. Afterwards, 1.5-mL tubes were packed separately and stored at −80°C until biochemical analysis. The plasma lipid metabolism parameters were determined as follows: total cholesterol (TCH; A111-2-1), triglyceride (TG; A110-2-1), creatinine (Cr; C011-2-1), low-density lipoprotein cholesterol (LDL-C; A113-2-1), and high-density lipoprotein cholesterol (HDL-C; A112-2-1). The plasma antioxidant activity parameters were determined as follows: TAC (A015-1-2), SOD (A001-1-2), GPX (A004-1-1), CAT (A007-1-1), 2,2-diphenyl-1-picrylhydrazyl (DPPH) free radical scavenging capacity (A153-1-1), MDA (A003-1-2), hydroxyl free radical (·OH; A018-1-1), and superoxide anion free radical (O_2_·; A052-1-1). All commercial kits were obtained from Nanjing Jiancheng Bioengineering Institute (Nanjing, China), and all plasma parameters were determined according to the manufacturers’ instructions.

### Carcass yield and relative organ weight

At the end of the experimental period and after blood collection, a total of 40 rabbits were slaughtered for carcass yield, relative organ weight, meat quality, fatty acid profile, and gene expression analyses. The carcass yield was detected as described by Li et al. (2022). Briefly, each rabbit was weighed before being slaughtered (preslaughter weight). Each rabbit was skinned and decapitated, and the skin was weighed (skin weight). Next, the stomach, trachea, intestines, reproductive organs, oesophagus, pancreas, spleen, and lungs were removed, with the kidneys, heart, and liver retained. Then, the carcass was weighed (half-bore weight). After that, the kidneys, heart, and liver were removed, and the carcass was weighed again (full-bore weight). The half-bore rate and full-bore rate were calculated according to the following equations: half-bore rate (%) = half-bore weight (g)/preslaughter weight (g) × 100; full-bore rate (%) = full-bore weight (g)/preslaughter weight (g) × 100.

The visceral organs, including the heart, liver, spleen, lungs, and kidneys, were separated and recorded. The relative weights of the heart, liver, spleen, lungs, and kidneys were calculated according to Yakubu et al. (2007) according to the following equation: relative organ weight (%) = fresh organ weight (g)/preslaughter weight (g) ×100.

### Meat quality

After being slaughtered, each rabbit carcass was stored at 4 °C for 24 h. Next, the LTL muscle was separated and postmortem measurements of muscle pH were taken at 24 h (pH_24h_) from the LTL using a portable pH meter (Matthäus, Eckelsheim, Germany). The colour parameters for lightness (L^*^), redness (a^*^), and yellowness (b^*^) of the LTL were detected using a portable meat colorimeter (NR110, Chongqing Qinyougong Technology Development Co., Ltd, Chongqing, China) at 24 h. Drip loss was calculated from slices of LTL suspended inside polythene bags and held at 4 °C for 24 h and was expressed as a percentage of initial weight. The meat sample was cut and weighed, covered with 17 layers of filter paper, and subjected to 30 kg of pressure for 5 min using a meat press machine (Tenovo Meat-1; Tenovo International Co., Limited Beijing, China). The water loss rate was expressed as a percentage of the initial weight. The LTL cores (1 × 3 cm) were obtained from the middle portion of the roasted sample by cutting them perpendicular to the fibre direction, and the shear force was detected using a Warner–Bratzler shear device (Xielikeji Co., Ltd, Harbin, China).

### Fatty acid profile

The individual fatty acid profile of LTL was analysed according to the method of the Chinese Standard GB 5009.168–2016 with minor modifications. Briefly, total lipids were extracted with a chloroform–methanol solution (2:1). Saponification was performed using a potassium hydroxide–methanol solution, and the products were converted to methyl esters using a 2% sodium hydroxide–methanol solution. The extraction mixture was injected into an Agilent 6890 gas chromatograph (GC; Agilent Technologies, Santa Clara, CA) equipped with a flame ionization detector and a polydicyanopropyl siloxane capillary column (100 m × 0.25 mm × 0.20 μm) with a 1 μL injection volume. The GC conditions were as follows: the initial column temperature was 100 °C, held for 13 min, increased to 180 °C at a rate of 10 °C/min, held for 6 min, increased to 200 °C at a rate of 1 °C/min, held for 10 min, increased to 230 °C at a rate of 4 °C/min, and held for 10.5 min. The injection port temperature was 270 °C, the detector temperature was 280 °C, the gas carrier was nitrogen, and the split ratio was 100:1. Peaks were identified by comparing the retention times, with glyceryl triundecanoate used as an internal standard. The amount of FA was expressed as a percentage of the total FA.

### Gene expression

Approximately 1 g of LTL muscle was immediately collected, transferred to a 1.5 mL tube, snap frozen in liquid nitrogen, and stored at −80°C until analysis. The expression of seven genes, namely, *Nrf2*, *Keap1*, haem oxygenase 1 (*HO1*), NAD(P)H quinone dehydrogenase 1 (*NQO1*), superoxide dismutase 1 (*SOD1*), glutathione peroxidase 1 (*GPX1*), and *CAT*, was detected. The housekeeping gene used in this study was glyceraldehyde-3-phosphate dehydrogenase (*GAPDH*; Table 3). Total RNA was extracted using the TRIzol method. The cNDA was synthesized using a commercial kit (Thermo Fisher Scientific, WI, USA). Next, real-time PCR was performed as follows: 1 μL of 10× diluted cDNA, 1 μL of forward primer, 1 μL of reverse primer, 5 μL of Bio-Rad Applied Biosystems PowerUp SYBR Green Master Mix, and 1 μL of distilled water. The cycling conditions were as follows: preincubation for 10 min at 95 °C, amplification for 30 s at 95 °C (40 cycles), and annealing for 1 min at 60 °C.

**Table 3.** Primer sequence information in this study

Gene^1^	Primer sequences (5′ to 3′)	Accession number	Product size, nt	Annealing temperature, °C
***Nrf2***	F: AAAGAAGGAAACGCCTGGGA	MK645905.1	166	60
	R: GAAGTCATCGACAGGGAGGT			
***Keap1***	F: CTGCAAGGACTACCTGGTGA	XM_008251549.4	139	60
	R: CAGGTAGCTGAGGGACTGG			
***HO1***	F: AACTTTCAGAAGGGCCAGGT	AY421756.1	110	60
	R: GGGTTCTCCTTGTTGTGCTC			
***NQO1***	F: AGGAAGGACATCACAGGCAA	XM_002711667.5	182	60
	R: AGAATGGCAGGGACTCCAAA			
***SOD1***	F: AGCCTGCTGGTTGTAGACAT	XM_070071880.1	102	60
	R: TCACCACAGGTACTGAAAGCA			
***GPX1***	F: TGCTGCTCATTGAGAATGTGG	NM_001085444.1	75	60
	R: CTTGCAGCTCGTTCATCTGG			
***CAT***	F: AGGGATGCCCTACTGTTTCC	XM_002709045.5	152	60
	R: GGAATCCCTCGGTCACTGAA			
***GAPDH***	F: GATCCCGCCAACATCAAGTG	DQ403051.1	79	60
	R: TCTCCATGGTGGTGAAGACG			

*Nrf2*, nuclear factor (erythroid-derived 2)-like 2; Keap1, kelch-like ECH-associated protein 1; *HO1*, haem oxygenase 1; NQO1, NAD(P)H quinone dehydrogenase 1; *SOD1*, superoxide dismutase 1; *GPX1*, glutathione peroxidase 1; *CAT*, catalase; *GAPDH*, glyceraldehyde-3-phosphate dehydrogenase; F, forward; R, reverse.

### Statistical analysis

The cage was considered the experimental unit for growth performance and nutrient utilization parameters, and a rabbit was considered the experimental unit for plasma parameters, carcass yield, relative organ weight, meat quality, fatty acid content, and gene expression. In addition, the relative mRNA gene expression data from the LTL muscle were calculated using the 2^−ΔΔCT^ method, and the control group data were considered the calibrator. All the parameters were analysed using SAS 9.1.3 software for one-way analysis of variance according to the following model: *y_ij_* = *μ  *+  *τ_i_* + *ε_ij_*, where *y_ij_* is an observation in treatment *i* (*i* = CON, LG, MG, and HG)*, μ* is the overall mean, *τ_i_* is the effect of treatment *i*, and *ε_ij_* is random error with a mean of 0 and variance of *σ*^2^. In addition, the mortality rate was analysed using a chi-square test. A statistically significant difference was considered at the level of *P *< 0.05.

## Results

### Growth performance

No significant differences (*P *> 0.05) in the ADFI or FCR were noted between rabbits receiving GLE supplementation and those in the CON group (Table 4). Compared with the CON treatment, the MG treatment resulted in a significantly (*P *< 0.05) greater BW at 42 d and significantly (*P *< 0.05) greater concentrations of BWC and ADG from 0–42 d. Furthermore, compared with the CON treatment, the LG treatment resulted in significantly (*P *< 0.05) greater BWC and ADG from 21–42 d.

**Table 4.** Effect of ginkgo leaf extract on growth performance in rabbit

Item^1^	Treatment^2^				SEM	*P*-value
	CON	LG	MG	HG		
**ADFI, g/d**						
**0 d–21d**	65.84	66.46	65.84	65.80	0.950	0.953
**21 d–42 d**	83.68	84.01	84.00	83.98	1.580	0.999
**0 d–42 d**	74.76	75.23	74.92	74.89	1.670	0.998
**BW, g**						
**0 d**	1068.06	1062.67	1070.47	1082.56	19.574	0.928
**21 d**	1466.94	1439.00	1503.59	1469.25	24.832	0.418
**42 d**	1862.75^b^	1925.27^ab^	1958.76^a^	1931.25^ab^	22.526	0.049
**BWC, g**						
**0 d–21d**	398.88	376.33	433.12	386.69	21.968	0.362
**21 d–42 d**	395.81^b^	486.27^a^	455.18^ab^	462.00^ab^	25.765	0.040
**0 d–42 d**	794.69^b^	862.60^ab^	888.29^a^	848.69^ab^	27.220	0.046
**ADG, g/d**						
**0 d–21d**	18.99	17.92	20.62	18.41	1.046	0.362
**21 d–42 d**	18.85^b^	23.16^a^	21.68^ab^	22.00^ab^	1.227	0.040
**0 d–42 d**	18.92^b^	20.54^ab^	21.15^a^	20.21^ab^	0.648	0.046
**FCR**						
**0 d–21d**	3.62	3.96	3.44	3.73	0.214	0.458
**21 d–42 d**	5.07	4.04	4.00	4.04	0.351	0.144
**0 d–42 d**	4.03	3.75	3.58	3.82	0.140	0.212

In the same row, values with different small superscript letters are significantly different (*P *< 0.05).

1 ADFI, average daily feed intake; BW, body weight; BWC, body weight change; ADG, average daily gain; FCR, feed conversion ratio.

2 CON, rabbit was fed a basal diet; LG, rabbit was fed a basal diet with 2 g/kg GLE; MG, rabbit was fed a basal diet with 4 g/kg GLE; HG, rabbit was fed a basal diet with 6 g/kg GLE.

### Mortality rate

The mortality rate was significantly lower (*P *< 0.05) in rabbits fed a diet supplemented with GLE than in those in the CON group (Table 5).

**Table 5.** Effect of ginkgo leaf extract on mortality rate in rabbit

Item	Treatment^1^				*x* ^2^-value	*P*-value
	CON	LG	MG	HG		
**Number of dead rabbits**	3	0	0	0	-	-
**Mortality rate, %**	15	0	0	0	9.351	0.029

1 CON, rabbit was fed a basal diet; LG, rabbit was fed a basal diet with 2 g/kg GLE; MG, rabbit was fed a basal diet with 4 g/kg GLE; HG, rabbit was fed a basal diet with 6 g/kg GLE.

### Nutrient utilization

No significant differences (*P *> 0.05) in the apparent digestibility of DM or NDF were noted among all the treatments (Table 6). However, the apparent digestibility of ADF, hemicellulose, and ash in the rabbits receiving MG was greater (*P *< 0.05) than that in the CON group. For nitrogen utilization, no significant difference (*P *> 0.05) in N intake was detected among the four treatments (Table 7). N excretion in faeces and total N excretion in the CON group were greater (*P *< 0.05) than those in the GLE treatment groups. In contrast, N absorption was significantly greater (*P *< 0.05) in the MG treatment than in the other three treatments. Additionally, compared with the CON group, dietary supplementation with GLE significantly (*P *< 0.05) increased N retention levels. With respect to energy utilization, no significant difference (*P *> 0.05) in GE intake was noted among the four treatments (Table 8). GE excretion in faeces, GE excretion in urine, and total GE excretion in the CON group were greater (*P *< 0.05) compared with those in the MG treatment group. In contrast, GE absorption and GE retention were significantly greater (*P *< 0.05) in the MG treatment compared with the other three treatments.

**Table 6.** Effect of ginkgo leaf extract on apparent digestibility in rabbit

Item, %^1^	Treatment^2^				SEM	*P*-value
	CON	LG	MG	HG		
**DM**	32.31	37.51	39.79	33.98	1.969	0.064
**NDF**	35.65	33.83	35.27	33.25	0.582	0.152
**ADF**	23.06^b^	20.88^b^	28.04^a^	20.16^b^	0.721	0.001
**Hemicellulose**	14.11^b^	24.60^a^	26.84^a^	25.62^a^	1.509	0.006
**Ash**	15.77^c^	19.76^b^	27.30^a^	20.61^b^	0.205	<0.001

In the same row, values with different small superscript letters are significantly different (*P *< 0.05).

1 DM, dry matter; NDF, neutral detergent fibre; ADF, acid detergent fibre.

2 CON, rabbit was fed a basal diet; LG, rabbit was fed a basal diet with 2 g/kg GLE; MG, rabbit was fed a basal diet with 4 g/kg GLE; HG, rabbit was fed a basal diet with 6 g/kg GLE.

**Table 7.** Effect of ginkgo leaf extract on nitrogen utilization in rabbit

Item^1^	Treatment^2^				SEM	*P*-value
	CON	LG	MG	HG		
**N intake, g/d**	1.94	1.95	1.95	1.95	0.002	0.052
**N excretion in feces, g/d**	0.44^b^	0.46^a^	0.38^c^	0.43^b^	0.003	<0.001
**N excretion in urine, g/d**	0.12^a^	0.08^b^	0.02^d^	0.04^c^	0.001	<0.001
**Total N excretion, g/d**	0.56^a^	0.54^b^	0.40^d^	0.47^c^	0.003	<0.001
**N absorption, g/d**	1.50^c^	1.49^bc^	1.57^a^	1.52^b^	0.004	<0.001
**N absorption, %**	77.42^b^	76.38^b^	80.46^a^	77.89^b^	0.189	<0.001
**N retention, g/d**	1.38^d^	1.42^c^	1.55^a^	1.47^b^	0.004	<0.001
**N retention, %**	71.18^d^	72.54^c^	79.47^a^	75.61^b^	0.170	<0.001

In the same row, values with different small superscript letters are significantly different (*P *< 0.05).

1 N, nitrogen.

2 CON, rabbit was fed a basal diet; LG, rabbit was fed a basal diet with 2 g/kg GLE; MG, rabbit was fed a basal diet with 4 g/kg GLE; HG, rabbit was fed a basal diet with 6 g/kg GLE.

**Table 8.** Effect of ginkgo leaf extract on energy utilization in rabbit

Item^1^	Treatment^2^				SEM	*P*-value
	CON	LG	MG	HG		
**GE intake, kJ/d**	1148.00	1155.27	1150.42	1149.97	4.207	0.809
**GE excretion in feces, kJ/d**	593.90^a^	600.45^a^	482.88^b^	586.94^a^	8.931	<0.001
**GE excretion in urine, kJ/d**	28.48^a^	17.92^b^	10.97^c^	10.01^c^	0.498	<0.001
**Total GE excretion, kJ/d**	622.38^a^	618.37^a^	493.85^b^	596.95^a^	9.030	<0.001
**GE absorption, kJ/d**	554.11^b^	554.82^b^	667.54^a^	563.03^b^	10.180	<0.001
**GE absorption, %**	48.27^b^	48.02^b^	58.03^a^	48.95^b^	0.816	<0.001
**GE retention, kJ/d**	525.63^b^	536.90^b^	656.57^a^	553.02^b^	10.306	<0.001
**GE retention, %**	45.79^b^	46.47^b^	57.08^a^	48.08^b^	0.828	<0.001

In the same row, values with different small superscript letters are significantly different (*P *< 0.05).

1 GE, gross energy.

2 CON, rabbit was fed a basal diet; LG, rabbit was fed a basal diet with 2 g/kg GLE; MG, rabbit was fed a basal diet with 4 g/kg GLE; HG, rabbit was fed a basal diet with 6 g/kg GLE.

### Plasma biochemical parameters

In terms of lipid metabolism parameters, the levels of TG, Cr, and HDL-C did not differ among the four groups (*P *> 0.05; Table 9). Compared with the CON group, the GLE groups presented significantly (*P *< 0.05) decreased plasma TCH content. Compared with the CON group, the LG group presented decreased (*P *< 0.05) plasma LDL-C levels. In terms of antioxidant activity, the treatments had no effect (*P *> 0.05) on the plasma SOD, GPX, or O_2_· values. Compared with CON, MG significantly increased (*P *< 0.05) the levels of TAC and DPPH scavenging activity. In addition, compared with the CON, supplementation with GLE significantly (*P *< 0.05) increased the plasma CAT level, whereas MG significantly (*P *< 0.05) decreased the MDA content. Compared with that of the CON and LG treatments, the plasma ·OH content of the rabbits decreased (*P *< 0.05) under the MG and HG treatments.

**Table 9.** Effect of ginkgo leaf extract on plasma lipid metabolism and antioxidant activity parameters in rabbit

Item^1^	Treatment^2^				SEM	*P*-value
	CON	LG	MG	HG		
**Lipid metabolism**						
**TCH, mmol/L**	2.20^a^	1.34^b^	1.74^b^	1.55^b^	0.133	0.001
**TG, mmol/L**	0.74	0.72	0.81	0.70	0.090	0.859
**Cr, μmol/L**	97.04	106.61	108.37	97.33	6.349	0.520
**LDL-C, mmol/L**	0.66^a^	0.48^b^	0.51^ab^	0.64^ab^	0.053	0.046
**HDL-C, mmol/L**	0.99	0.81	0.87	0.98	0.087	0.411
**Antioxidant activity**						
**TAC, U/mL**	1.87^b^	2.20^b^	4.28^a^	2.47^b^	0.294	0.013
**SOD, U/mL**	104.09	104.55	102.70	105.86	1.334	0.423
**GPX, U/mL**	26.82	30.92	26.18	26.32	2.283	0.426
**CAT, U/mL**	1.51^b^	2.81^a^	3.85^a^	3.66^a^	0.387	<0.001
**DPPH scavenging activity, %**	58.66^b^	65.74^ab^	76.32^a^	64.81^ab^	3.451	0.018
**MDA, nmol/mL**	2.49^a^	1.52^b^	1.02^b^	1.46^b^	0.266	0.044
**·OH, U/mL**	974.68^a^	981.29^a^	592.84^b^	322.61^c^	59.659	<0.001
**O_2_·, U/L**	335.20	339.92	338.15	336.38	2.910	0.683

In the same row, values with different small superscript letters are significantly different (*P *< 0.05).

1 TCH, total cholesterol; TG, triglyceride; Cr, creatinine; LDL-C, low-density lipoprotein cholesterol; HDL-C, high-density lipoprotein cholesterol; TAC, total antioxidant capacity; SOD, superoxide dismutase; GPX, glutathione peroxidase; CAT, catalase; DPPH, 2,2-diphenyl-1-picrylhydrazyl; MDA, malondialdehyde; ·OH, hydroxyl free radical; O_2_·, superoxide anion free radical.

2 CON, rabbit was fed a basal diet; LG, rabbit was fed a basal diet with 2 g/kg GLE; MG, rabbit was fed a basal diet with 4 g/kg GLE; HG, rabbit was fed a basal diet with 6 g/kg GLE.

### Carcass yield and relative organ weight

No significant differences (*P *> 0.05) in the carcass yield parameters, including preslaughter weight, skin weight, half-bore weight, half-bore rate, full-bore weight, or full-bore rate, were noted among the four treatments (Table 10). Similarly, the weights of the heart, liver, spleen, lung and kidney of rabbits receiving GLE did not differ (*P *> 0.05) compared with those in the CON group.

**Table 10.** Effect of ginkgo leaf extract on carcass yield and relative organ weight in rabbit

Item	Treatment^1^				SEM	*P*-value
	CON	LG	MG	HG		
**Carcass yield**						
**Pre-slaughter weight, g**	1684.60	1689.10	1651.50	1658.80	19.711	0.454
**Skin weight, g**	126.65	119.78	127.51	117.13	4.060	0.210
**Half-bore weight, g**	1072.30	1075.59	1026.63	1016.54	20.472	0.130
**Half-bore rate, %**	63.96	63.75	62.13	60.55	1.173	0.210
**Full-bore weight, g**	818.39	783.99	794.20	776.84	20.377	0.506
**Full-bore rate, %**	48.24	46.40	48.09	46.82	1.216	0.641
**Visceral organs, %**						
**Heart**	0.33	0.29	0.27	0.27	0.028	0.330
**Liver**	2.54	2.61	2.51	2.41	0.059	0.130
**Spleen**	0.070	0.072	0.056	0.066	0.005	0.109
**Lung**	0.70	0.59	0.60	0.62	0.033	0.089
**Kidney**	0.58	0.55	0.55	0.55	0.021	0.593

In the same row, values with different small superscript letters are significantly different (*P *< 0.05).

1 CON, rabbit was fed a basal diet; LG, rabbit was fed a basal diet with 2 g/kg GLE; MG, rabbit was fed a basal diet with 4 g/kg GLE; HG, rabbit was fed a basal diet with 6 g/kg GLE.

### Meat quality

No significant differences (*P *> 0.05) in the values of pH_24h_ or lightness in muscle were noted among all the treatments (Table 11). The redness values were greater (*P *< 0.05) in rabbits receiving MG compared with those receiving the other three treatments. Compared with those fed the CON diet, rabbits fed the LG and MG diets presented decreased (*P *< 0.05) levels of yellowness, drip loss, and shear force in the muscle.

**Table 11.** Effect of ginkgo leaf extract on meat quality in rabbit

Item	Treatment^1^				SEM	*P*-value
	CON	LG	MG	HG		
**pH_24h_**	5.87	5.82	5.93	5.89	0.032	0.157
Lightness, *L* ^*^	49.17	46.09	45.75	46.42	1.034	0.094
Redness, *a* ^*^	2.08^b^	2.96^b^	4.79^a^	1.80^b^	0.440	<0.001
Yellowness, *b* ^*^	9.08^a^	6.50^b^	7.32^b^	6.19^b^	0.466	<0.001
**Drip loss, %**	3.53^a^	2.41^b^	2.48^b^	3.10^ab^	0.227	0.010
**Shear force, N**	21.86^a^	18.42^b^	19.00^b^	19.55^ab^	0.939	0.039

In the same row, values with different small superscript letters are significantly different (*P *< 0.05).

1 CON, rabbit was fed a basal diet; LG, rabbit was fed a basal diet with 2 g/kg GLE; MG, rabbit was fed a basal diet with 4 g/kg GLE; HG, rabbit was fed a basal diet with 6 g/kg GLE.

### Fatty acid profile

No significant differences (*P *> 0.05) in the muscle C15:0, C16:1, C18:2n6c, C18:3n6, C20:5n3, C22:5n-3, n-6 PUFA, or PUFA concentrations were noted among the four treatments (Table 12). Compared with the CON diet, the addition of GLE significantly (*P *< 0.05) decreased C16:0 and C20:4n6 levels, whereas it significantly (*P *< 0.05) increased C18:3n3 and n-3 PUFA levels. Compared with the CON group, the MG treatment tended to increase (*P *< 0.05) C14:0, C18:1n9c, MUFA, and C22:6n3 levels, whereas the MG treatment significantly decreased (*P *< 0.05) the C20:3n6 content. The C17:0 levels of the LG and HG treatments were greater (*P *< 0.05) than those of the CON and MG treatments. Compared with those in the LG and MG groups, the C18:0 and SFA levels in the HG treatment significantly increased (*P *< 0.05).

**Table 12.** Effect of ginkgo leaf extract on fatty acid profiles in rabbit

Item^1^, %	Treatment^2^				SEM	*P*-value
	CON	LG	MG	HG		
**C14:0**	0.75^c^	1.14^ab^	1.36^a^	1.12^b^	0.067	0.001
**C15:0**	0.67	0.67	0.66	0.62	0.017	0.162
**C16:0**	25.99^a^	23.18^bc^	22.97^c^	23.73^b^	0.222	<0.001
**C17:0**	0.69^b^	0.90^a^	0.57^b^	0.91^a^	0.042	0.001
**C18:0**	8.61^b^	9.60^b^	8.64^b^	11.30^a^	0.403	0.005
**SFA**	36.74^ab^	35.52^bc^	34.20^c^	37.67^a^	0.481	0.005
**C16:1**	0.61	0.57	0.65	0.51	0.076	0.648
**C18:1n9c**	20.38^b^	21.38^b^	23.81^a^	20.64^b^	0.506	0.005
**MUFA**	21.00^b^	21.94^b^	24.46^a^	21.15^b^	0.519	0.005
**C18:2n6c**	28.60	29.74	31.04	29.55	0.507	0.054
**C18:3n6**	0.26	0.25	0.23	0.23	0.013	0.304
**C18:3n3**	1.32^c^	1.93^b^	2.26^a^	1.79^b^	0.055	<0.001
**C20:3n6**	0.67^a^	0.63^a^	0.51^b^	0.63^a^	0.029	<0.001
**C20:4n6**	10.80^a^	9.32^b^	6.53^d^	8.33^c^	0.162	<0.001
**C20:5n3**	0.17	0.18	0.18	0.17	0.021	0.978
**C22:5n-3**	0.31	0.37	0.41	0.33	0.046	0.502
**C22:6n3**	0.11^b^	0.14^ab^	0.19^a^	0.15^ab^	0.021	0.043
**n-3 PUFA**	1.94^c^	2.60^b^	3.04^a^	2.44^b^	0.052	<0.001
**n-6 PUFA**	40.32	39.94	38.30	38.74	0.640	0.161
**PUFA**	42.26	42.54	41.34	41.18	0.625	0.389

In the same row, values with different small superscript letters are significantly different (*P *< 0.05).

1 SFA, saturated fatty acids; MUFA, monounsaturated fatty acids; PUFA, polyunsaturated fatty acid.

2 CON, rabbit was fed a basal diet; LG, rabbit was fed a basal diet with 2 g/kg GLE; MG, rabbit was fed a basal diet with 4 g/kg GLE; HG, rabbit was fed a basal diet with 6 g/kg GLE.

### Gene expression

**Figure 1.**
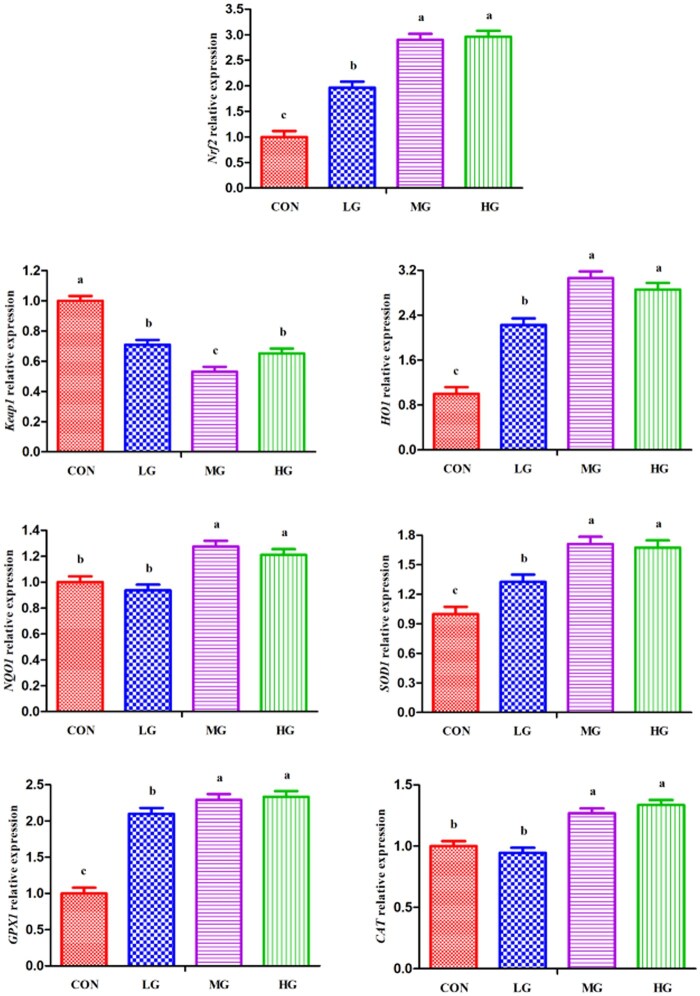
The relative expression of antioxidant genes in the *longissimus thoracis et lumborum* muscle in rabbits. Data were reported as mean ± SEM. The relative mRNA gene expression data from the LTL muscle was calculated by the 2^-ΔΔCT^ method, and the CON group data were considered as the calibrator. Different small letters are significantly different (*P *< 0.05). *Nrf2*, nuclear factor (erythroid-derived 2)-like 2; Keap1, kelch-like ECH-associated protein 1; *HO1*, haem oxygenase 1; *NQO1*, NAD(P)H quinone dehydrogenase 1; *SOD1*, superoxide dismutase 1; *GPX1*, glutathione peroxidase 1; *CAT*, catalase; CON, rabbit was fed a basal diet; LG, rabbit was fed a basal diet with 2 g/kg GLE; MG, rabbit was fed a basal diet with 4 g/kg GLE; HG, rabbit was fed a basal diet with 6 g/kg GLE.

Compared with those in the CON group, the levels of *Nrf2*, *HO1*, *SDO1*, and *GPX1* in the LTL muscle in the GLE groups significantly increased (*P *< 0.05; Figure 1). In addition, rabbits receiving the MG and HG diets had significantly increased (*P *< 0.05) *NQO1* and *CAT* mRNA levels related to the CON and LG groups. In contrast, the addition of GLE to the rabbit diet significantly decreased (*P *< 0.05) *Keap1* mRNA levels in LTL muscle compared with the CON group.

## Discussion

In this study, we aimed to determine the effects of GLE on growth performance, nutrient utilization, antioxidant activity, carcass yield, meat quality, fatty acid profiles, and gene expression in rabbits. The results indicated that the inclusion of 4 g/kg GLE can improve growth performance, nutrient utilization, meat quality, and antioxidant activity in growing rabbits.

### Growth performance

A previous study reported the consumption of 150 g/kg flavonoid-rich bilberry pomace decreased the FCR of Grimaud rabbits (Dabbou et al., 2019). Moreover, Hassan et al. (2016) reported that the addition of 0.3 g/kg grape seed extract increased body weight gain and decreased the FCR of weaned male New Zealand White rabbits. In the current study, we found that compared with the CON group, 4 g/kg GLE increased the growth performance of rabbits, and this effect is potentially related to greater nutrient utilization (apparent digestibility, N balance and energy balance) in the 4 g/kg GLE group (Tables 6–8). A similar study conducted by Elwan et al. (2019) revealed that dietary supplementation with 2% flavonoid-rich *Citrus limon* powder increased the final weight and ADG and decreased the FCR value in growing rabbits. However, the inclusion of a high dose of GLE (6 g/kg) did not affect the growth performance of the rabbits. This is possibly this may be because the rabbits did not have a significantly different DMI, which would potentially suggest poor palatability for high levels of bioactive compounds in the diets of small ruminants (da Silva et al., 2017).

### Mortality rate

Weaned rabbits are prone to OS because of nutritional dyspepsia and dysfunction of the gut microbiota, which results in increased morbidity and mortality rates (Gidenne and Fortun-Lamothe, 2004; Khalifa et al., 2024). In addition, Wang et al. (2025) reported that dietary supplementation with plant extract could increase antioxidant activity parameters and subsequently reduce the mortality rate in weaned rabbits. In the present study, GLE decreased the mortality rate of rabbits, possibly because flavonoid compounds in plants can inhibit the growth of pathogenic bacteria, improve the gastrointestinal microbiota composition and gut immune function, promote gastrointestinal development and improve health, and thus decrease the mortality rate in rabbits (Liu et al., 2019; Benlemlih et al., 2020; Abou-Kassem et al., 2025). Our observations are consistent with those of Hassan et al. (2016), who reported that dietary supplementation with 0.3 g/kg grape seed extract could reduce the mortality rate in growing rabbits.

### Nutrient utilization

Apparent digestibility is typically used to evaluate the efficiency of nutrient utilization in animal feed and is an important indicator of animal feed utilization capacity (Kiarie et al., 2014). In the present study, the inclusion of 4 g/kg GLE increased the apparent digestibility of ADF, hemicellulose, and ash, indicating that an appropriate level of GLE improved nutrient digestibility in rabbits. This improvement in nutrient digestibility potentially occurred because flavonoid compounds result in the modulation of the gut microbiota and improve the immune response in animals (Ahmed and Yang, 2017). Dietary supplementation with flavonoid-rich citrus extract improved the absorption and utilization of nitrogen in piglets (Cui et al., 2020). In the present study, we reported that dietary supplementation with 4 g/kg GLE increased N absorption and N retention in growing rabbits. This potentially occurred because flavonoids bind to CP (nitrogen), forming metastable aggregates and thus improving nitrogen utilization in animals (Bordenave et al., 2014; Zhan et al., 2017). Short-chain fatty acids (SCFAs) synthesized by the gut microbiome are a direct source of energy for rabbits and affect the energy metabolism of their host (Deng et al., 2025). Gao et al. (2025) reported that dietary supplementation with 15% flavonoid-rich kudzu vine (*Pueraria lobata*) meal increased caecal acetic acid, propionic acid, and total SCFA production in meat rabbits. Notably, most flavonoids present in plants are bound to sugars such as b-glycosides, and glycans can be easily absorbed by the intestine (Kumar and Pandey, 2013). In the present study, we found that supplementation with 4 g/kg GLE improved the energy balance of rabbits, indicating that flavonoids could improve energy utilization in rabbits. This may be because flavonoids are antioxidants present in plant feeds and occur mainly as glycans, and these glycans might participate in SCFA metabolism in rabbits (Kumar and Pandey, 2013; Jurgoński et al., 2014; Khalid et al., 2022) and subsequently improve the energy balance. However, this conclusion needs to be further confirmed in the future. In brief, the inclusion of 4 g/kg GLE improved nutrient utilization (apparent digestibility, N and energy balance) in rabbits.

### Lipid metabolism parameters

Flavonoid compounds promote the breakdown and oxidation of fat by regulating the expression of genes related to fat metabolism, thereby reducing fat accumulation (Ouyang et al., 2016). In addition, flavonoid compounds inhibit the proliferation and differentiation of adipocytes, reduce the number of adipocytes, and thus regulate lipid metabolism parameters in animals (Rufino et al., 2021). Li et al. (2014) reported that dietary supplementation with flavonoid extract improved lipid metabolism parameters by decreasing total cholesterol in the blood and by downregulating adipose differentiation-related protein expression in the liver of animals. In the present study, the addition of GLE decreased TCH and LDL-C levels in the plasma of rabbits. The reason for this may be that flavonoid compounds prevent hepatic steatosis by reducing SREBP1c-mediated lipogenesis and cholesterol acyltransferase activity, resulting in reduced plasma TG and cholesterol concentrations in rabbits (Jeon et al., 2004; Assini et al., 2013). Another mechanism of action involves the ability of flavonoid compounds to block specific transporters for cholesterol (Yuberro et al., 2013). Consistent with our observations, Al-Samarrai et al. (2017) reported that the administration of bay leaves and their isolated flavonoids and glycosides reduced TC, TG, LDL-C and VLDL-C serum levels in female Iraqi rabbits.

### Antioxidant activity parameters

The antioxidant and oxidative defence systems are in dynamic equilibrium in the animal body under normal physiological conditions (Pamplona and Costantini, 2011). However, weaned rabbits are prone to OS because the physiological balance of oxidants and antioxidants is disrupted (Chen et al., 2022a). Vizzarri et al. (2020) reported that dietary supplementation with flavonoid-rich plants positively affects plasma antioxidant markers and thus enhances the antioxidant status of weaned rabbits. In the present study, we found that dietary supplementation with GLE improved plasma TAC, CAT, and DPPH scavenging activity while reducing MDA and ·OH concentrations in growing rabbits. The following reasons are proposed: (1) Flavonoids have strong antioxidant activity, mainly because of their multiple phenolic hydroxyl groups; the hydroxyl groups have a strong redox ability and can react with free radicals, thereby reducing damage from free radicals to biomolecules (Cao et al., 1997). (2) Flavonoids can enhance antioxidant effects by inhibiting the activity of oxidases and reducing the production of free radicals in rabbits (Dabbou et al., 2018). (3) Flavonoids inactivate metal ions and inhibit pro-oxidant enzymes, thereby reducing the generation of free radicals catalysed by metal ions and delaying lipid peroxidation (Sugihara et al., 1999). Consistent with our results, Elwan et al. (2019) reported that the inclusion of 2% flavonoid-rich *Citrus limon* powder increased blood TAC, SOD, GSH, GST, and CAT concentrations while reducing blood MDA levels in growing New Zealand White rabbits.

### Carcass yield and relative organ weight

In the present study, dietary supplementation with GLE did not affect the carcass yield or relative organ weight of the rabbits, possibly because the nutritional composition of each group was consistent, except for the different levels of GLE added. Our results indicated that GLE does not cause adverse reactions to carcass yield and does not influence production performance. These findings are in agreement with the observations of Dabbou et al. (2018), who reported that dietary alfalfa flavonoids had no adverse effects on carcass yield or relative organ weight parameters in crossbred rabbits. The normal development of internal organs is an important foundation and prerequisite for the operation and physiological function of animal bodies and reflects the health conditions of animals (Smith et al., 2011). Moreover, visceral organ size is related to dry matter intake (DMI), as the energy consumption of these organs increases after feeding and is dependent on the DMI (Zhang et al., 2017). In the present study, GLE did not affect the relative visceral organ weight, suggesting that dietary supplementation with GLG had no adverse effects on the organ indices of meat rabbits. The possible reason may be that there was no significant difference in the DMI between rabbits in the GLE group and those in the CON group.

### Meat quality

Meat colour is among the most intuitive indicators for evaluating meat quality and plays important roles in marketing and consumer acceptability (Ruedt et al., 2023). Moreover, lipid oxidation can result in deterioration of the colour, taste, and texture of meat products and damage their nutritional value (Kumar et al., 2013). Interestingly, flavonoid compounds are the active ingredients of ginkgo leaves and exhibit a variety of biological activities, including the inhibition of free radical-scavenging and lipid peroxidation properties (van Beek, 2002). Thus, a basal diet supplemented with 4 g/kg GLE could reduce the L^*^ value and increase the a^*^ value, possibly because flavonoid compounds act as strong antioxidants that inhibit haem autoxidation, thereby decreasing the L^*^ value and increasing the a^*^ value in muscle (Xie et al., 2022). Drip loss and shear force are important parameters of muscle tenderness, and high values indicate low tenderness (Wyrwisz et al., 2012). In addition, flavonoid compounds may improve water retention performance, which is very beneficial for maintaining tenderness, juiciness, and taste and improving meat quality (Serra et al., 2021). Xie et al. (2022) reported that the inclusion of 3 g/kg flavonoid-rich olive leaf extract decreased the shear force of breast meat in broiler chickens. In the present study, we found that dietary supplementation with 2 g/kg and 4 g/kg GLE decreased the values of drip loss and shear force, indicating that GLE could improve LTL taste and increase the degree of LTL delicacy in rabbits. However, the mechanisms by which GLE maintains tenderness are unclear and deserve further investigation. Moreover, further studies of sensory properties are needed to evaluate our findings. Our observations were in agreement with those of Perna et al. (2019), who reported that dietary intake of 30% flavonoid-rich cauliflower leaf powder decreased drip loss and cooking loss values but increased L^*^ and a^*^ values in the LTL muscle of growing rabbits. Moreover, Wang et al. (2023) reported that dietary supplementation with 300 mg/kg yucca extract could decrease the shear force and increase meat tenderness in weaned New Zealand White rabbits. Interestingly, compared with the CON group, the high dose of GLE (6 g/kg) did not affect the drip loss or shear force. This may be related to the phenolic hydroxyl groups in high levels of flavonoids binding to feed starch molecules, increasing the interaction of hydrogen bonding networks, and decreasing viscosity, thereby increasing drip loss and shear force values (Zheng et al., 2025).

### Fatty acid profile

The PUFAs are cell membrane components that are important nutrients, have various physiological functions, have been broadly investigated and have received much attention from the public (Harwood, 2023). However, highly oxidizable PUFAs are prone to lipid oxidation reactions in animal meat, which can negatively affect meat quality (Ponnampalam et al., 2012; Adeyemi et al., 2022a). Dietary supplementation with natural antioxidants improved the antioxidant activity of rabbit meat and subsequently prevented oxidative deterioration and maintained the PUFA concentration (Adeyemi et al., 2022b). Flavonoid compounds have good resistance to lipid peroxides and can retard lipid peroxidation in muscle, thereby helping to maintain the PUFA content in animals (Nguyen et al., 2017). Hence, flavonoids may increase the fatty acid composition of meat through the inhibition of PUFA oxidation in rabbits (Selim et al., 2021). Liu et al. (2022) reported that the inclusion of flavonoid compounds from mulberry leaves could increase the concentrations of C18:3n3 and n-3 PUFAs in the *longissimus lumborum* muscle of finishing pigs. Similar results were also recorded in meat rabbits when 2% linseed oil plus 0.5% green tea powder was included in the diets of growing Californian male rabbits (Eid et al., 2010). In the present study, we found that the dietary addition of GLE improved the muscle n-3 PUFA content in rabbits. An explanation for these observed effects on fatty acid composition is that flavonoid compounds can increase the expression levels of genes encoding factors related to fatty acid β-oxidation as well as fatty acid synthesis (Tan et al., 2022) and thus inhibit PUFA oxidation and maintain PUFA contents in rabbits. Consistent with our findings, Selim et al. (2021) reported that dietary supplementation with 1.5 g/kg flavonoid-rich *Moringa oleifera* leaves increased the relative content of n-3 PUFA in the meat of growing New Zealand white rabbits. Similarly, North (2019) reported that dietary supplementation with 2 g/kg quercetin dehydrated C18:3n-6, C20:3n-6, C20:3n-3 and C20:4n-6 in muscle and decreased the n-6: n-3 ratio in New Zealand White rabbits.

### Gene expression

Antioxidative enzymes can remove excessive amounts of free radicals and other harmful substances, thereby alleviating the OS status in animals (Bešlo et al., 2023). Notably, Nrf2 is critical for cellular defence against OS and can induce endogenous increases in the levels of the antioxidant enzymes, antioxidant proteins, and anti-inflammatory and detoxification proteins, all of which can play a protective role in animals (Wu et al., 2022). However, Keap1 inhibits Nrf2 activity and maintains an inactive state in the cytoplasm (Stępkowski and Kruszewski, 2011). Previous studies have shown that the dietary addition of flavonoid-rich plant extract can increase the activity of the antioxidant defence system through the activation of the Nrf2 signalling pathway and the expression of antioxidant genes, ultimately enhancing the antioxidant function in animals (Gessner et al., 2017; Shi et al., 2024). This occurred because antioxidants cause Nrf2 to dissociate from Keap1 and thus increase the abundance of downstream antioxidant genes (e.g., SOD, GPX, and CAT) in animals (Wang et al., 2025). Chen et al. (2022b) reported that dietary supplementation with flavonoid-rich plant extract could alleviate OS status by upregulating Nrf2 and antioxidant genes and downregulating Keap1 in animals. Thus, the results of the current study revealed that the dietary addition of moderate (4 g/kg) and high (6 g/kg) doses of flavonoid-rich GLE increased *Nrf2* gene expression; increased the expression of its downstream antioxidant genes *HO1*, *SDO1*, and *GPX1*; and decreased the abundance of Keap1 mRNA in rabbits. These findings correspond to the plasma antioxidant activity parameters (Table 9). This might be because flavonoids promote Nrf2 nuclear translocation and subsequently upregulate the expression and activity of Nrf2 and its downstream antioxidant genes, ultimately reducing OS damage and enhancing antioxidant function in animals (Lu et al., 2022). However, a low dose of (2 g/kg) flavonoid-rich GLE did not affect the gene expression of *NQO1* or *CAT* in rabbits, perhaps because low levels of natural phenolic antioxidants do not sufficiently increase antioxidant activity (Pokorný, 2007). In brief, it is hypothesized that dietary supplementation with GLE might increase the antioxidant function of activating the Nrf2 signalling pathway, ultimately contributing to the alleviation of OS status and enhancing antioxidant activity and immune function in rabbits.

## Conclusion

The results of the present study suggest that GLE exhibits good potential as a natural antioxidant in rabbit feed because (1) it can increase BWC, ADG, and nutrient utilization; (2) it can improve plasma lipid metabolism and antioxidant activity parameters; (3) the intake of GLE can improve meat quality and n-3 PUFA levels; and (4) the addition of GLE can upregulate the abundance of *Nrf2* and its downstream antioxidant genes. Overall, under the conditions of this experiment, the optimum supplementation level of GLE in the diets of growing rabbits was 4 g/kg. However, we did not elucidate the molecular mechanism underlying the enhancement of antioxidant performance by GLE, and further studies based on transcriptomics and proteomics are needed to determine the mechanism by which GLE improves antioxidant capacity. In addition, GLE contains various flavonoid compounds, and the effects of pure flavonoids and their possible interactions with muscle n-3 PUFAs need to be studied further.

## Abbreviations

*a* ^*^: redness
ADF: acid detergent fibre
ADFI: average daily feed intake
ADG: average daily gain
*b* ^*^: yellowness
BW: body weight
BWC: body weight change
CAT: catalase
CP: crude protein
Cr: creatinine
DM: dry matter
DPPH: 2,2-diphenyl-1-picrylhydrazyl
FCR: feed conversion ratio
GE: gross energy
GPX: glutathione peroxidase
GPX1: glutathione peroxidase 1
HDL-C: high-density lipoprotein cholesterol
HO1: haem oxygenase 1
Keap1: kelch-like ECH-associated protein 1
*L* ^*^: lightness
LDL-C: low-density lipoprotein cholesterol
MDA: malondialdehyde
MUFA: monounsaturated fatty acids
N: nitrogen
NDF: neutral detergent fibre
NQO1: NAD(P)H quinone dehydrogenase 1
Nrf2: nuclear factor (erythroid-derived 2)-like 2
O_2_·: superoxide anion free radical
^·^OH: hydroxyl free radical
OM: organic matter
PUFA: polyunsaturated fatty acid
SCFA: short-chain fatty acid
SFA: saturated fatty acid
SOD: superoxide dismutase
SOD1: superoxide dismutase 1
TAC: total antioxidant capacity
TCH: total cholesterol
TG: triglyceride
